# Psychometric properties of the Positive Mental Health Scale (PMH-scale)

**DOI:** 10.1186/s40359-016-0111-x

**Published:** 2016-02-10

**Authors:** Justina Lukat, Jürgen Margraf, Rainer Lutz, William M. van der Veld, Eni S. Becker

**Affiliations:** Mental Health and Treatment Center, Ruhr-University Bochum, Massenbergstraße 9-13, 44787 Bochum, Germany; University of Marburg, Marburg, Germany; Behavioural Science Institute, Radboud University Nijmegen, Nijmegen, The Netherlands

## Abstract

**Background:**

In recent years, it has been increasingly recognized that the absence of mental disorder is not the same as the presence of positive mental health (PMH). With the PMH-scale we propose a short, unidimensional scale for the assessment of positive mental health. The scale consists of 9 Likert-type items.

**Methods:**

The psychometric properties of the PMH-scale were tested in a series of six studies using samples from student (*n* = 5406), patient (*n* = 1547) and general (*n* = 3204) populations. Factorial structure and measurement equivalence were tested with the measurement invariance testing. The factor models were analysed with the maximum likelihood procedure. Internal consistency was examined using Cronbach’s alpha, test-retest reliability, convergent and divergent validity was examined by Pearson correlation. Sensitivity to (therapeutic) change was examined with the *t*-test.

**Results:**

Results confirmed unidimensionality, scalar invariance across samples and over time, high internal consistency, good retest-reliability, good convergent and discriminant validity as well as sensitivity to therapeutic change.

**Conclusions:**

These findings suggest that the PMH-Scale indeed measures a single concept and allows us to compare scores over groups and over time. The PMH-scale thus is a brief and easy to interpret instrument for measuring PMH across a large variety of relevant groups.

## Background

Mental health has traditionally been defined as the absence of psychopathology [30]: Individuals were seen as either mentally ill or presumed to be mentally healthy. In recent years, however, it is increasingly recognized that the absence of mental disorder is not the same as the presence of positive mental health [62]. Thus, elements of positive mental health (PMH) and mental health problems can be present at the same time: They are seen as independent but correlated concepts (e.g., [31, 36, 52]). In this view, both positive mental health (PMH, often also referred to as mental well-being) and mental disorder (often referred to as mental health problems, psychopathology or negative well-being) are required for complete mental health assessments and should be integrated in research (“dual-factor model of mental health”, e.g., [52])^1^.

Two theories dominate the field regarding the components of PMH [9, 45]: The hedonic tradition deals with positive affect (or positive emotions and moods) and high life-satisfaction, whereas the eudaimonic tradition focuses on optimal functioning of an individual in everyday life [29, 30, 60]. Taking both the hedonic and the eudaimonic approaches into account, PMH can be defined as the presence of general emotional, psychological, and social well-being [32]. While individual characteristics of PMH can be measured with specific instruments (e.g., [14, 50, 53]), comprehensive questionnaires assess multiple dimensions of PMH (e.g., Mental Health Continuum-Short Form MHC-SF; [33]) or include items relating to both PMH and psychopathology (e.g., General Health Questionnaire GHQ; [22]). The Positive Mental Health Scale (PMH-scale; Lutz et al. 1992a, unpublished manuscript) was developed to measure positive mental health with a brief, unidimensional and person-centred questionnaire. Unidimensionality [51] ensures that the scale measures a single concept as postulated by the holistic concept of PMH. Person-centred items have the advantage that statements consist of cross-situationally stable judgments about the participant rather than predictions about specific behaviours in particular situations (“I am…”, e.g., Freiburg Personality Inventory FPI; [12]).

Several criteria for the formulation and content of the items were used for the initial selection of the items for the PMH-scale: The items had to correspond with the definition of PMH being general, cross-situational and person-centred. The person-centred items focus on the consistency of a person’s overall characteristic pattern across many situations, while the behaviour-centred items instead focus on the behaviour pattern in specific situations [15]. In addition, the PMH-scale was constructed to measure the inner factors (e.g. emotional and psychological) of positive mental health in suppose to the outer factors (e.g. social support, partnership). The scale was to require having an unidimensional, self-reporting, brief, easy to complete and sensitive to change. For the development of the PMH-scale, Lutz et al. (1992a, unpublished manuscript) used items from their own item pool and from the pool of four German language instruments that met these selection criteria (Trier Personality Inventory, [5]; Freiburg Personality Inventory, [12]; Mental Health Scale, [53]; Bernese questionnaire of subjective well-being, [17]). The final version that Lutz et al. (1992a, unpublished manuscript) produced was reduced to nine items (see Table 1) that are rated on a Likert scale from 1 (*not true*) to 4 (*true*).

**Table 1:** The items of the PMH-scale and their origin

	Item	Origin of the item
1.	I am often carefree and in good spirits.	Trierer Personality Inventory (TPF)
2.	I enjoy my life.	Item from Lutz’s item pool
3.	All in all, I am satisfied with my life.	Freiburg Personality Inventory (FPI-R)
4.	In general, I am confident.	Mental health scale (SPG)
5.	I manage well to fulfill my needs.	Trier Personality Inventory (TPF)
6.	I am in good physical and emotional condition.	Trier Personality Inventory (TPF)
7.	I feel that I am actually well equipped to deal with life and its difficulties.	Trier Personality Inventory (TPF)
8.	Much of what I do brings me joy.	Mental Health Scale (SPG)
9.	I am a calm, balanced human being.	Trier Personality Inventory (TPF)

Earlier version of the PMH-scale was found to be among the most important predictors of remission from specific [54] or social phobia [59] in the Dresden Predictor Study of Mental Health [55]. Analyses of a broad range of predictors of remission from specific phobia [54] of 137 participants revealed that protective factors, particularly *positive mental health* and *life satisfaction* at baseline, were predictive of remission. Other protective (*social support* and *self-efficacy*), vulnerability factors (*twelve-month stress, coping skills, negative cognitive style* and *psychopathology*) and specific phobia characteristics (*severity* and *age of onset*) at baseline did not predict course of specific phobia. Recovery from social phobia [59] of 91 participants was significantly predicted by less *psychopathology*, less *anxiety sensitivity*, less number and less *stress of daily hassles* and better *positive mental health*. In a multivariate regression model, after adjustment of the other salient predictors of recovery, positive mental health showed to be the strongest predictor of recovery from social phobia. These results support health promotion programs focused on salutogenetic factors and not only prevention concerning traditional pathogenetic factors and mental disorders. In addition, the PMH-scale and the wish to receive pension were found (Lutz and Michalak 2001, unpublished manuscript) to predict the success of behaviour therapy with inpatients.

**Fig. 1:**
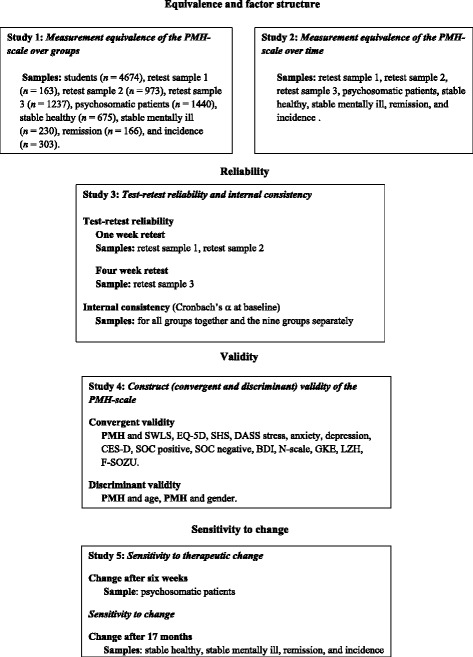
An overview over the five studies – objectives and samples

These results encouraged us to investigate the psychometric properties and usefulness of the current nine-item version of the PMH-scale in greater detail. For this purpose we conducted a series of five studies building on samples drawn from patients (*n* = 1547), students (*n* = 5406) and the general population with and without mental disorders (*n* = 3204). This study had the following objectives (Fig. 1):

- To examine whether the nine items of the PMH-scale load on a single factor and to examine the equivalence of the PMH-scale for different populations (study 1).
- To examine whether the PMH-scale is invariant over time (study 2); a requirement for usage in intervention studies.
- To estimate the test-retest reliability, internal consistency and correlation across time of the scale (study 3).
- To establish the construct validity of the scale by assessing its convergent and discriminant validity (study 4).
- To evaluate the scale’s sensitivity to therapeutic change (study 5).

## Methods

### Samples and procedures

Table 2 shows the socio-demographic data for all samples. Participants in all samples had given informed consent based on information about the individual studies and the assurance of anonymity. The studies form part of the larger Bochum Optimism and Mental Health study program (BOOM). The ethics committee approval was different for the samples. The ethical approval for the student sample and the retest samples was obtained by the Ethics Committee of the Faculty of Psychology at Ruhr-University Bochum. The study with the patient sample received ethical approval from the German Federal Insurance Institution for Employees (Bundesversicherungsanstalt für Angestellte; BfA). The study with the Dresden sample received ethical approval from the Office for Data Protection (in Saxony, Amt für Datenschutz, Staat Sachsen) and the State of Saxony Public Health Association.

**Table 2:** Socio-demographic data for the seven samples (percentage of the respective samples)

Characteristics	Students	Retest samples			Patients	Dresden Predictor Study samples			
		Retest sample 1	Retest sample 2	Retest sample 3		Stable healthy	Incidence	Stable mentally ill	Remission
Sample size	5406	167	1004	1294	1547	683	169	232	310
Age (years): mean (S.D.)	26.3 (4.0)	36.7 (12.9)	43.4 (13.0)	54.7 (17.1)	48.9 (8.4)	22.7 (1.8)	22.6 (1.8)	22.7 (1.8)	22.7 (1.8)
Gender (%)									
Female	55.5	68.9	49.9	43.9	74.8	100	100	100	100
Male	44.5	31.1	50.1	56.1	25.2	-	-	-	-
Marital status (%)									
Single	30.3	12.6	34.6	21.6	8.3	35.0	40.2	37.1	35.2
Partnership	40.0	36.5	2.1	1.1	7.9	60.6	56.8	57.8	59.7
Married	0.3	43.7	51.7	57.0	62.8	4.2	3.0	5.2	4.8
Divorced	0.3	6	9.9	9.1	15.7	0	0	0	0
Widowed	0	1.2	1.7	11.2	5.3	0.1	0	0	0.3
Occupation (%)									
Self-employed	-	6.7	-	-	2.7	0.3	1.4	1.1	1.4
Manual worker/technician	-	8.6	-	-	9.3	12.5	13.7	16.0	13.7
Simple/mid-level employee	-	66.9	-	-	61.5	51.4	47.9	54.3	68.3
White-collar/executive employee	-	4.3	-	-	13.6	4.2	2.7	4.3	5.8
Other (students, pensioner etc.)	100	13.5	20.5	39.4	12.7	31.6	34.2	24.5	24.5
Employment (%)									
Full time	-	66.5	47.7	32.0	45.3	29.6	26.6	27.6	28.7
Part time	-	25.1	18.4	17.25	21.3	17.7	15.4	15.5	19.0
Non-working	-	8.4	6.8	8.0	15.9	50.4	54.4	49.1	46.8
Unemployed	-	0	6.5	3.3	17.5	2.4	3.6	7.8	5.5

#### Student sample

In the fall of 2011, all 31.994 students of Ruhr-University Bochum received an e-mail inviting them to participate in a survey on mental health. Based on information about the study and an assurance of anonymity, a total of 5406 students (16.9 %) gave informed consent and participated in the survey that consisted of a demographic questionnaire, the PMH-scale, self-report instruments (some of which are used to validate the PMH-scale, e.g. SWLS; EQ-5D; SHS; DASS stress, anxiety, depression; SOZU-K) and five additional questionnaires that are not analysed in this study.

#### Retest sample 1

Participants were recruited in March and April 2012 among employees of three different facilities (St.Elisabeth Hospital Hattingen, Lebenshilfe Kleve, Tagesklinik Warstein) and inhabitants of the city of Kleve (Germany). Via a snowball sampling procedure we invited acquaintances, current and former colleagues per e-mail or a letter, to participate in the study. Based on information about the study and an assurance of anonymity, 167 participants, neither seeking mental health care nor receiving psychological treatment gave informed consent and completed a battery of questionnaires consisting of a demographic questionnaire, the PMH-scale and three other self-report instruments used to validate the PMH-scale (CES-D; SOC; N-scale). After an average of 7.4 days, 138 participants (83 %) completed the PMH-scale a second time. There were no significant differences between completers and dropouts at baseline. To minimize the administrative burden, the SOC and the CES-D were included in the baseline survey and the N-scale was included in the follow-up.

#### Retest samples 2 and 3

In summer and fall 2013, two samples representative for the German adult population (age 18 and above) were recruited via telephone. Participants completed the PMH-scale online or by mail twice with a time lag of either one week (retest sample 2, *n* = 1004) or four weeks (retest sample 3, *n* = 1294).

#### Patient sample

Between January 1998 and August 2000 data was collected on 1547 patients who received cognitive-behavioral therapy in the psychosomatic hospital “Edertal” in Bad Wildungen (Germany). After giving informed consent patients completed a battery of questionnaires within one to six days after their admission at the clinic. The average treatment time was six weeks. One to five days before discharge, 80 % (*n* = 1232) of the patients who had participated at the baseline completed the questionnaires for the second time. The diagnoses of the patients were based on the standard diagnostic criteria according to the ICD-9 definitions [61]. At baseline, 25.3 % (*n* = 391) of the 1547 patients were missing data on the main diagnosis. Out of the 1156 patients with a main diagnosis 60.3 % had neurotic disorders, 17.4 % a functional disorder of psychological origin, 17 % an adjustment disorder, 1.6 % an affective psychosis, and 3.7 % another diagnosis. A total of 56.4 % of the patients had at least one comorbid diagnosis.

#### Dresden sample

This sample consisted of 1394 young German women who participated in the Dresden Predictor Study (DPS; [55]), a prospective epidemiological study of mental disorders. In the DPS, young women aged 18–25 years were randomly selected from the 1996 population registers of residents of Dresden (Germany). A baseline survey was conducted from July 1996 to September 1997 and a follow-up assessment 17 months later (*M* = 16.9 months, *SD* = 6.0, range = 7–30 months). At both times, structured clinical interviews for DSM-IV diagnoses were conducted with each participant (F-DIPS; translation: Research Diagnostic Interview for Psychological Disorders, [39]; this is the German version of the Anxiety Disorder Interview Schedule-Lifetime, ADIS-IV-L; DiNardo et al. 1995). The anxiety disorders included generalized anxiety disorder, panic disorder with and without agoraphobia, agoraphobia without history of panic disorder, specific phobia, social phobia, obsessive-compulsive disorder, posttraumatic stress disorder and acute stress disorder. The affective disorders included dysthymic disorder, major depressive disorder (single episode, recurrent), bipolar I and II disorder and cyclothymic disorder. The somatoform disorders included somatization disorder, undifferentiated somatoform disorder, conversion disorder, pain disorder and hypochondriasis. The substance disorders included alcohol, medicine and drug abuse and dependency. The eating disorders included anorexia nervosa and bulimia nervosa. The current study is restricted to those participants who completed the diagnostic interview as well as a battery of self-report questionnaires including the PMH-scale at both times of data collection. For the determination of sensitivity to change of the PMH-scale we divided the Dresden sample into four subgroups based on the absence or presence of mental disorders at the two assessment times as follows:

#### Stable healthy

Participants (*n* = 683) who had no history of a mental disorder and who had no mental disorder at any time.

#### Incidence

Participants (*n* = 166) who did not suffer from any disorder prior to the baseline or at the baseline, but who developed one or more mental disorders during the 17-month follow-up period. At follow-up, 75.1 % of these women suffered from anxiety disorder, 27.8 % from affective disorder, 5.9 % from somatoform disorder, 3.6 % from substance abuse and/or dependence and 2.4 % from eating disorder.

#### Stable mentally ill

Participants (*n* = 232) who met DSM-IV criteria for a lifetime prevalence of one or more mental disorders at the first assessment and who also suffered from a mental disorder at the second assessment (baseline/follow-up diagnoses: Anxiety disorder: 79.7 % / 81.5 %, affective disorder: 32.8 % / 27.6 %, somatoform disorder: 6.9 % / 8.2 %, substance abuse and/or dependence: 16.9 % / 8.2 %, eating disorder: 8.6 % / 7.3 %, childhood mental disorders: 21.6 % / 0 %).

#### Remission

Participants (*n* = 310) who met criteria for at least one mental disorder only in the past and/or at baseline, but not at the follow-up. At the initial assessment 59.7 % of these women had an anxiety disorder, 31.6 % an affective disorder, 6.8 % a somatoform disorder, 1.9 % a substance abuse and/or dependence, 9.7 % an eating disorder and 24.5 % a lifetime prevalence of childhood mental disorders.

### Instruments

All studies employed the PMH-scale. In addition, the following instruments were used in study 4 to determine convergent and discriminant validity^2^:

- Social Support Scale (SOZU-K; [16]). Higher scores indicate greater levels of social support.
- Satisfaction With Life Scale (SWLS; [11]). High scores denote high levels of satisfaction.
- EuroQol Health Questionnaire (EQ-5D; [42]). Low scores point to good subjective health.
- Subjective Happiness Scale (SHS; [37]). Higher scores essentially reflect higher levels of subjective happiness.
- Depression Anxiety Stress Scales-21 (DASS-21; [34]). Higher scores mark greater levels of distress (stress, anxiety, and depression).
- Neuroticism (N-scale, a modified version of the 12-item emotionality scale of the revised Freiburg Personality Inventory, FPI-R; [12]). Participants scored the items on a scale from 1 (not true) to 4 (true) in the modified version instead of a 2-point rating scale. High scores on the N-scale indicate high neuroticism.
- Sense of Coherence Scale (SOC; [35]). High scores on the SOC point to a high sense of coherence.
- Center for Epidemiological Studies Depression Scale (CES-D; German version; [43]; German: Allgemeine Depressionsskala Kurzform, ADS-K; [18]). Higher scores indicate pronounced levels of depressive symptoms.
- General Self-efficacy Scale (GKE; [27]). Higher scores denote more self-efficacy.
- Life Satisfaction Questionnaire (LZH; Lutz et al. 1992b, unpublished manuscript). Lower scores indicate higher life satisfaction.
- Beck Depression Inventory (BDI; [4]). High scores signify more severe depression.

In study 5 (sensitivity to therapeutic change) the following instrument was used in addition to the PMH-scale:

- Global Assessment of Functioning (GAF; [2]). This numeric 0-100 scale is used by mental health clinicians to rate the social, occupational, and psychological functioning of adults. High scores represent a high level of functioning.

### Statistical analyses

The PMH-scale is a general scale designed to measure PMH in a variety of groups on single occasions and across time. Therefore, it is required that the PMH-scale is equivalent for groups and across time. Measurement equivalence is tested with a procedure called measurement invariance testing [41]. Three forms of measurement invariance are important for the goals that we intend to use our PMH-scale for: (1) configural, (2) metric, and (3) scalar invariance. The test for configural invariance determines whether the factor structure is the same over groups and/or across time. In the test for metric invariance we determine whether the scale of the latent factor (PMH-scale) has the same metric over groups and/or across time. This is tested by imposing equality constraints on factor loadings. The scalar invariance test establishes whether the scale of the latent factor (PMH-scale) has the same zero point over groups and/or across time. This is tested by imposing equality constraints on the item intercepts.

It is important for a scale to have these invariance properties, because they have consequences for the interpretation of the analyses with this scale, such as comparing groups. When a scale is configural invariant, the same construct is measured over groups and/or across time. In case a scale is metric invariant, it is valid to compare relations with other variables over groups and/or across time. If a scale is scalar invariant, means over groups and/or across time can be compared. This is particularly important when the scores of the PMH-scale are to be compared between groups and/or across time.

We analysed the factor models with LISREL 8.8 [28] using the maximum likelihood procedure. This procedure was applied to the data even though the variables were not normally distributed (skewness ranged between -1.6 and 1.0 and the kurtosis ranged between -1.0 and 2.4). However, robustness studies by Anderson and Amemiya [1], Satorra and Bentler [49], and Satorra [48] have shown that the so-called “quasi maximum likelihood” estimator, which is LISREL’s implementation of ML, is robust under quite general conditions.

Current practice to evaluate model fit is to use fit indices, especially the RMSEA. Recent studies, however, have shown that fit indices with fixed critical values (e.g., the RMSEA, GFI) do not work as intended because it is not possible to control for type I and type II errors [3, 40, 47]. In this study, the number of observations is very high, resulting in very high power to detect even the smallest misspecification. As a result our model will be rejected, even though it is adequate for all practical purposes. An alternative procedure to evaluate models was developed by Saris et al. [47]. They suggest searching for misspecifications in the model. Hu and Bentler [21] state that a model is misspecified when (a) one or more parameters are estimated while their population values are zero, (b) one or more parameters are fixed to zero while their population values are not zero, and (c) both. Saris et al. consider the second type of misspecification the most serious. In their procedure they combine the modification indices (indicating the second type of misspecifications discussed above) with the power of the test to detect misspecifications. This procedure is implemented in the software package JRule (Van der Veld et al. [57]). For the current multigroup factor analyses we used the JRule settings as described in Van der Veld and Saris [56]. Model evaluation in this procedure entails testing for misspecified parameters. If misspecified parameters are found, they are either estimated in the model or their equality constraints are removed. Despite the fact that one single misspecification may invalidate the interpretation of the model, we do accept that models may have several misspecifications. This is in line with Browne and Cudeck [7] and MacCallum et al. [38] who suggested that models are always simplifications of reality and are therefore always misspecified. Therefore, we were rather sparse with model modifications. We do aim for a factor model that represents a scale that is adequate for all practical purposes. That means that we ignore a (misspecified) parameter if estimation of that parameter hardly changes the interpretation of the model. In our factor analyses we have operationalized hardly as when factor loadings change less than approximately .07 after the introduction of a (misspecified) parameter. In addition to this new model evaluation procedure we do also provide the RMSEA, the NNFI, and the *χ*^2^ test statistic for the reader who is interested in those figures.

Finally, the search for misspecifications was focused on different parameters in the tests for configural, metric, and scalar invariance. In the test for configural invariance, the model is constrained on the correlated errors; they are zero across the groups. Therefore we focused on misspecifications on that part - correlated errors - of the model. In the test for metric invariance the factor loadings are constrained; for each item they are the same across the groups. Therefore we focused on misspecifications on that part - factor loadings - of the model. In the test for scalar invariance the intercepts are constrained; for each items the intercepts are the same. Therefore we focused on misspecifications on that part - item intercepts - of the model. The word focus is used in the above sentences to mean more or less exclusively focus. Thus, misspecifications solved in the configural invariance test are first correlated errors, misspecifications solved in the metric invariance test are first factor loadings, and misspecifications solved in the scalar invariance test are first item intercepts. All other statistical analyses were conducted using IBM SPSS Statistics Version 21.0 [23].

## Results

### Descriptive statistics

Means (*M*), standard deviations (*SD*), skewness, and kurtosis were calculated for each of the nine PMH- scale items of the three largest samples (Table 3). The means of the 4-point Likert items ranged in the patient sample from 1.62 (Item 7) to 2.78 (Item 4), with average *M* of 2.36 and a *SD* of 0.33. The means ranged in the student sample from 2.81 (Item 5) to 3.19 (Item 4), with average *M* of 3.01 and a *SD* of 0.15. The means ranged in the Dresden sample from 2.77 (Item 9) to 3.60 (Item 9), with average *M* of 3.30 and a *SD* of 0.25.

**Table 3:** Means, standard deviations, skewness, kurtosis, factor loadings, item-total score-corrected correlations of the PMH-scale (the three largest samples)

Item		*M*	*SD*	Skewness	Kurtosis	Factor loading	r_tt_
Patient sample (N = 1547)							
1.	I am often carefree and in good spirits	2.30	0.84	0.31	-0.43	.78	.71
2.	I enjoy my life.	2.32	0.87	0.24	-0.59	.82	.76
3.	All in all, I am satisfied with my life.	2.48	0.97	-0.01	-0.99	.72	.64
4.	In general, I am confident.	2.78	0.90	-0.30	-0.70	.78	.71
5.	I manage well to fulfill my needs.	2.33	0.83	0.21	-0.48	.75	.67
6.	I am in good physical and emotional condition.	2.29	0.89	0.17	-0.74	.75	.67
7.	I feel that I am actually well equipped to deal with life and its difficulties.	1.62	0.76	1.02	0.31	.63	.55
8.	Much of what I do brings me joy.	2.37	0.93	0.08	-0.88	.80	.73
9.	I am a calm, balanced human being.	2.75	0.89	-0.13	-0.83	.78	.71
PMH-scale		2.36	0.66	.23	-0.51	-	-
Student sample (N = 5406)							
1.	I am often carefree and in good spirits	2.89	0.84	-0.33	-0.55	.81	.75
2.	I enjoy my life.	3.13	0.80	-0.65	-0.06	.84	.78
3.	All in all, I am satisfied with my life.	3.12	0.87	-0.70	-0.32	.84	.79
4.	In general, I am confident.	3.19	0.80	-0.72	-0.08	.82	.76
5.	I manage well to fulfill my needs.	2.81	0.80	-0.30	-0.33	.77	.71
6.	I am in good physical and emotional condition.	2.93	0.89	-0.46	-0.57	.82	.76
7.	I feel that I am actually well equipped to deal with life and its difficulties.	3.01	0.82	-0.50	-0.34	.82	.76
8.	Much of what I do brings me joy.	3.16	0.79	-0.61	-0.32	.81	.75
9.	I am a calm, balanced human being.	2.83	0.87	-0.33	-0.58	.64	.57
PMH-scale		3.00	0.66	-0.54	-0.30	-	-
Dresden sample (N = 1394)							
1.	I am often carefree and in good spirits	3.21	0.72	-0.60	0.10	.71	.61
2.	I enjoy my life.	3.48	0.65	-1.08	0.83	.71	.61
3.	All in all, I am satisfied with my life.	3.45	0.70	-1.20	1.17	.78	.70
4.	In general, I am confident.	3.48	0.66	-1.14	1.06	.75	.66
5.	I manage well to fulfill my needs.	3.10	0.66	-0.41	0.41	.69	.60
6.	I am in good physical and emotional condition.	3.26	0.73	-0.77	0.31	.74	.65
7.	I feel that I am actually well equipped to deal with life and its difficulties.	3.31	0.71	-0.84	0.51	.78	.70
8.	Much of what I do brings me joy.	3.60	0.60	-1.39	1.64	.74	.65
9.	I am a calm, balanced human being.	2.77	0.88	-0.31	-0.61	.52	.43
PMH-scale		3.3	0.50	-0.95	0.94	-	-

*M* mean, *SD* standard deviations, r_tt =_ Item-Total Score-Corrected Correlations

For the patient sample the scores revealed a reasonably normal distribution with the means for skewness and kurtosis being 0.18 (*SD* = 0.37) and -0.59 (*SD* = 0.38), respectively. None of the items had a skew or a kurtosis greater than 1 (in absolute value). For the student sample the scores revealed a reasonably normal distribution with the means for skewness and kurtosis being -0.51 (*SD* = 0.16) and -0.34 (*SD* = 0.18), respectively. None of the items had a skew or a kurtosis greater than 1 (in absolute value). In the Dresden samples the means for skewness and kurtosis being -0.87 (*SD* = 0.35) and 0.64 (*SD* = 0.64). Items 2, 3, 4 and 8 had a skew and items 3, 4, 8 had a kurtosis greater than 1 (in absolute value).

### Study 1: Measurement equivalence of the PMH-scale over groups

**Fig. 2:**
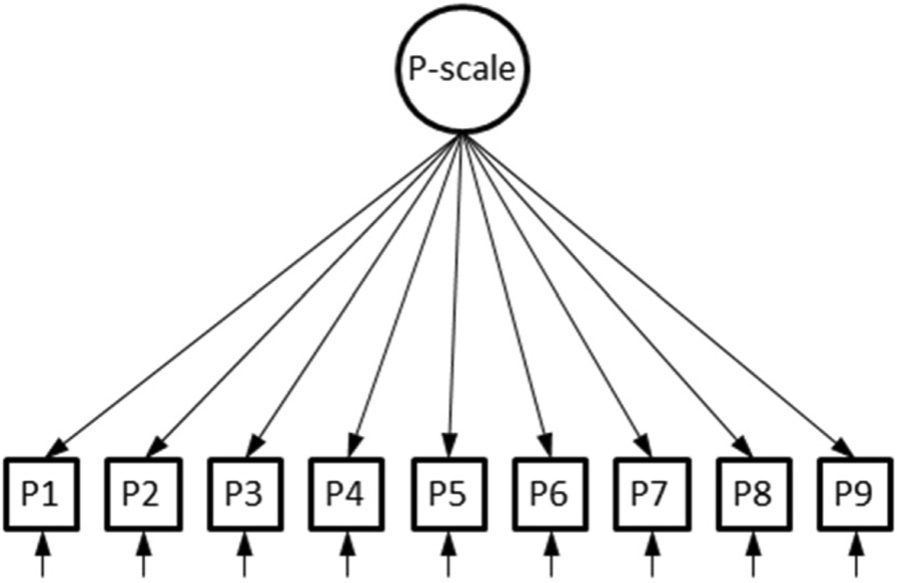
Path diagram of the measurement model of the PMH-scale

The factorial invariance of the PMH-scale was evaluated for the following nine groups: students (*n* = 4674), retest sample 1 (*n* = 163), retest sample 2 (*n* = 973), retest sample 3 (*n* = 1237), psychosomatic patients (*n* = 1440), stable healthy (*n* = 675), incidence (*n* = 303), stable mentally ill (*n* = 230), and remission (*n* = 166). We analysed the covariance matrices for the nine items measured at the first occasion. Figure 2 depicts the factor model that is tested for the nine groups.

The test for configural invariance resulted in a total of 32 misspecifications (out of a total of 324 possible misspecifications). We accepted this model without further modifications (*χ*^2^ = 2027.05, *df* = 243, RMSEA = 0.082, NNFI = 0.98, 32 misspecifications remaining). Next we tested the model with the restrictions for metric invariance; that is we added equality constraints across the groups on the factor loadings of the same items. The analysis resulted in 35 misspecifications (out of 388 possible misspecifications). We released one of the factor loading constraints in the group patients. After re-analysing the data we found 32 misspecifications (out of 388 possible misspecifications). We accepted this model without additional modifications (*χ*^2^ = 2270.09, *df* = 306, RMSEA = 0.077, NNFI = 0.98, 32 misspecifications remaining). Finally, we tested for the scalar invariance of the PMH-scale, that is we added equality constraints across the groups on the item intercepts of the same items. This resulted in 61 misspecifications (out of a total of 460 possible misspecifications). We released eleven item intercepts in nine groups. We accepted the resulting model without further modifications (*χ*^2^ = 2712.22, *df* = 358, RMSEA = 0.078, NNFI = 0.98, 33 misspecifications remaining). In conclusion, the PMH scale shows configural invariance, partial metric invariance and partial scalar invariance. Partial invariance [8] refers to the fact that we had to release several invariance restrictions during the testing process. Byrne and colleagues suggest that we can make valid inferences about relationships between factors and about the differences between latent factor means in the model when there are at least two factor loadings and item intercepts that are constrained equal across groups. However, if there is partial invariance (metric or scalar) then composite scores should not be used, since they will bias substantive conclusions [46]. Bias, on the other hand, is a matter of degree. The more severe the model deviates from full invariance, the larger the potential for bias. In this study, we found 1 factor loading and 12 item intercepts to be invariant. This is relatively low to the total number of constraints that still hold, i.e. 149 (80 factor loadings and 70 items intercepts are still invariant). In the light of these numbers, we do not think that the degree of invariance will seriously bias our conclusions. Therefore we will treat the PMH scale as if it was fully metric and scalar invariant in the subsequent studies.

### Study 2: Measurement equivalence of the PMH-scale across time

**Fig. 3:**
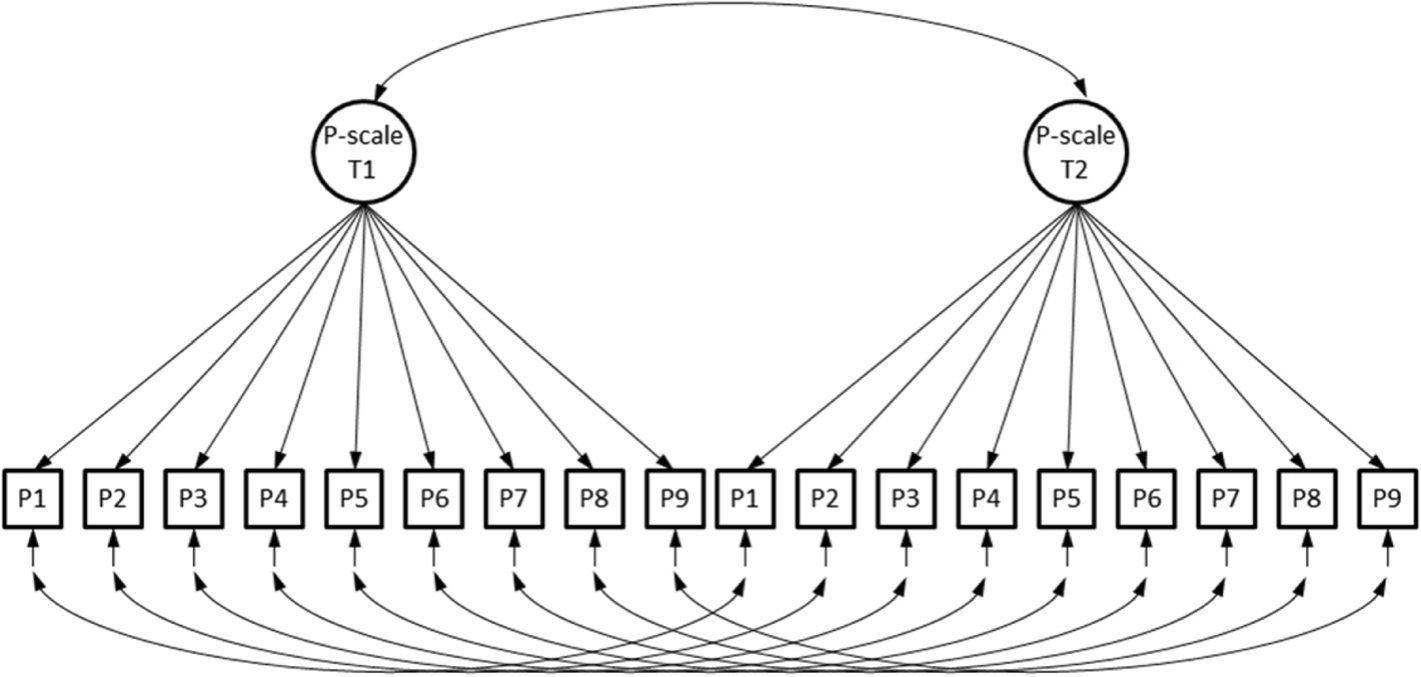
Path diagram of the two-wave measurement model of the PMH-scale

We concluded (study 1) that the partial invariance of the PMH scale should not seriously threaten the validity of conclusions resulting from treating the scale as if it was fully metric and fully scalar invariant. Therefore we will analyze the pooled data. We do not have a follow-up measurement for the students; therefore students were not included in the analysis. The number of observations for this analysis is 4750 after listwise deletion. We analysed the covariance matrices for the nine items measured at the first and second assessment occasion. The model that we analysed is depicted in Fig. 3. The model shows two factors that represent the PMH-scale at the first and second measurement occasion. We introduced correlated errors for the same items across time.

We estimated and tested the model to evaluate the factorial invariance of the PMH-scale across time. The analysis resulted in 4 misspecifications (out of 162 possible misspecifications). We accepted this model (*χ*^2^ = 2582.25, *df* = 125, RMSEA = 0.064, NNFI = 0.97, 4 misspecifications remaining). Next we tested the model with the restrictions for metric invariance, that is, we added equality constraints across time on the factor loadings of the same items. The analysis resulted in 3 misspecifications (out of 170 possible misspecifications) and we thus accepted the model (*χ*^2^ = 2599.56, *df* = 133, RMSEA = 0.062, NNFI = 0.97, 3 misspecifications remaining). Finally, we tested for scalar invariance of the PMH-scale across time, that is, we added equality constraints across time on the item intercepts of the same items. The analysis resulted in 3 misspecifications (out of 179 possible misspecifications); hence we accepted the model without further modifications (*χ*^2^ = 2458.43, *df* = 141, RMSEA = 0.061, NNFI = 0.98, 3 misspecifications remaining). In conclusion, the PMH-scale is invariant across time and therefore can be used validly compare PMH-scale scores across time. This is a requirement when sensitivity to change is studied.

### Study 3: Test-retest reliability and internal consistency

A change in the score on the PMH-scale should be the result of actual changes in the level of PMH and not the result of random measurement error. A high level of reliability is thus required. We estimated both the test-retest reliability as well as the internal reliability [44]. The test-retest reliability requires two measures in a short period of time; so short that one cannot expect change, but long enough that one cannot expect recall effect [58]. For the retest samples 1 and 2, the time between the repeated measures was one week, which is short enough to assess the test-retest reliability. For retest sample 3, the time interval was 4 weeks. In addition, we also have retest data available for other groups in this study and estimated the across time correlation. In those instances, however, the time between the repeated measures is not short enough to assume perfect stability. Therefore one can expect change across time and in that case the test-retest correlation indicates (in)stability as well as reliability. To estimate the test-retest reliability, we computed a mean score of the nine items of the PMH-scale at each measurement occasion. The correlation between the mean scores is an estimate of the reliability.

#### Test-retest reliability

The Pearson correlation between the first and second administrations (one week apart) of the PMH-scale in retest samples 1 and 2 was estimated (Table 4). The test-retest reliability of the PMH-scale was found to be .81 (*p* < .01) in retest sample 1 and .77 (*p* < .001) in retest sample 2. With a time lag of four weeks (retest sample 3), a test-retest reliability of .74 resulted (*p* < .001). Thus, the test-retest reliability is good.

**Table 4:** Test retest reliability, internal consistency and across time correlation of the PMH-scale

Samples		Test-retest reliability	Internal consistency	Across time correlation
	*n* ^a^	*r*	*α*	*r*
Students	4674	-	.93	-
Retest sample 1	138	.81**	.82	-
Retest sample 2	941	.77**	.91	-
Retest sample 3	1194	.74**	.90	-
Psychosomatic patients	1440	-	.91	.40**
Dresden sample – stable mentally healthy	683	-	.84	.57**
Dresden sample – incidence	166	-	.87	.57
Dresden sample – stable mentally ill	232	-	.90	.66**
Dresden sample – remission	310	-	.85	.57**
All groups	7652	-	.93	-

^a^n is the maximum number of participants in the analyses. The number of participants varied for each analysis

**p < .01

#### Internal consistency

Values for Cronbach’s alpha were estimated for the first occasion only (Table 4). The estimates were respectively: .93 for all groups together, .93 for the students, .82 for retest sample 1, .91 for retest sample 2, .90 for retest sample 3, .91 for the psychosomatic patients, .84 for the stable healthy, .87 for the incidence group, .90 for the stable mentally ill, and.85 for the remission group. The internal consistency of the PMH-scale was thus high and similar across different samples.

#### Across time correlation

The across time correlation between the first and second administrations of the PMH-scale in the psychosomatic patients sample and the four groups of the Dresden sample was estimated (Table 4). The across time correlation of the PMH-scale was found to be .40 (*p* < .01) in psychosomatic patients with a time lag of six weeks. The across time correlation was found to be .57 (*p* < .01) in the stable mentally healthy group, .57 (*p* > .01) in the incidence group, .66 (*p* < .01) in the stable mentally ill group and .57 (*p* < .01) in the remission group, with a time lag of 17 months for all four groups.

### Study 4: Construct (convergent and discriminant) validity of the PMH-scale

We assessed the convergent validity of the PMH-scale with the measures described in the section ‘Instruments’. The measures EQ-5D, DASS stress, DASS anxiety, DAS depression, CES-D, SOC negative, N-scale and BDI were expected to correlate negatively with the PMH-scale and the measures SWLS, SHS, SOC positive, GKE, LZH and SOZU-K were expected to correlate positively with the PMH-scale. We expected no correlations with these variables age and gender.

Table 5 presents the Pearson correlations between the PMH-scale and the other measures. All correlations are quite strong, supporting the construct validity of the PMH-scale. In addition, the correlations are in the expected direction, conditional on the positive or negative coding of the variables. For example satisfaction with life (SWLS) correlates positively with the PMH-scale (*r* = .75) because high scores on the SWLS indicate more satisfaction. Finally, we assessed the discriminant validity using the variables age and gender. We estimated the correlations between age – PMH-scale and gender – PMH-scale. As expected, those variables did not significantly correlate with the PMH-scale. This was not true for the students (*r* = .09; *r* = .07): In the case of the students, however, the sample size (*n* = 4667) is so large that even small correlations become significant.

**Table 5:** Pearson correlations of the PMH-scale with other scales at baseline

Scales	PMH-scale								
	Students	Retest sample 1	Retest sample 2	Retest sample 3	Patients	Stable healthy	Incidence	Stable mentally ill	Remission
*SWLS*	.75^*^	-	-	-	-	-	-	-	-
*EQ-5D*	-.59^*^	-	-	-	-	-	-	-	-
*SHS*	.81^*^	-	-	-	-	-	-	-	-
*DASS stress*	-.56^*^	-	-	-	-	-	-	-	-
*DASS anxiety*	-.51^*^	-	-	-	-	-	-	-	-
*DASS depression*	-.74^*^	-	-	-	-	-	-	-	-
*CES-D*	-	-.57^*^	-	-	-.71^*^	-	-	-	-
*SOC positive*	-	.26^*^	-	-	.59^*^	-	-	-	-
*SOC negative*	-	-.44^*^	-	-	-.61^*^	-	-	-	-
*N-scale*	-	-.50^*^	-	-	-.65^*^	-.53^*^	-.56^*^	-.64^*^	-.58^*^
*BDI*	-	-	-	-	-	-.53^*^	-.48^*^	-.68^*^	-.64^*^
*GKE*	-	-	-	-	-	.52^*^	.53^*^	.65^*^	.52^*^
*LZH*	-	-	-	-	-	.48^*^	.59^*^	.58^*^	.58^*^
*SOZU-K*	.52^*^	-	-	-	-	.49^*^	.52^*^	.57^*^	.52^*^
*Age*	-.09^*^	.00	.09*	.05	.04	.04	.06	.04	-.07
*Gender*	.07^*^	.10	.09*	-.01	-.04	-	-	-	-

* *p* < .05

### Study 5: Sensitivity to (therapeutic) change

The PMH-scale should be able to detect changes in PMH across time. We already established (study 2) that the PMH-scale is scalar invariant across time, which is necessary for *unbiased* comparisons across time. Traditionally change across time is assessed with a paired samples *t*-test. We will use the 4 groups in the Dresden sample and the sample of psychosomatic patients to study sensitivity to therapeutic change. The psychosomatic patients administered the PMH-scale twice with an interval of six weeks while receiving behaviorally oriented inpatient therapy. In the Dresden sample all participants completed the PMH scale twice within an interval of approximately 17 months. At both administrations the participants were classified with a structured clinical interview for DSM-IV diagnoses. Participants, who were diagnosed with a disorder, only very rarely searched for treatment.

Table 6 shows that PMH improved for all groups, however, the improvement for the groups in the Dresden sample is very modest, to say the least. The psychosomatic patients improved their PMH significantly, *t*(1230) = 17.51, *p* = .00, after 6 weeks of treatment. The effect size is moderate (*Cohen’s d* = .50). The improvement in PMH of the psychosomatic patients is corroborated by a simultaneous change in global health. Global health was measured with the GAF scale. There was a significant change in the GAF score between the first and second administration, *t*(561) = 24.40, *p* = .00). The effect size was large (*Cohen’s d* = .80).

**Table 6:** Change across time of the PMH-scale

	*n*	*M*_t1_ (*SD*)	*M*_t2 (*SD*)_	*M*_t2_-*M*_t1_	*SE*_*M*t2-*M*t1_
Psychosomatic patients	1231	2.35 (.66)	2.62 (.69)	.27**	.015
Dresden sample – stable mentally healthy	683	3.42 (.42)	3.49 (.39)	.07**	.014
Dresden sample – incidence	169	3.27 (.48)	3.29 (.50)	.02	.035
Dresden sample – stable mentally ill	232	2.97 (.60)	3.05 (.62)	.08*	.033
Dresden sample – remission	310	3.28 (.46)	3.37 (.42)	.08**	.023

*M* mean, *SD* standard deviations, *SE* standard error

* *p* < .05, **p < .01

The stable healthy improve significantly, *t*(682) = 4.92, *p* = .00, with a small effect size (*Cohen’s d* = .19). The stable mentally ill improve significantly, *t*(231) = 2.47, *p* = .01, with a small effect size (*Cohen’s d* = .16). The incidence group does not improve significantly, *t*(168) = 0.56, *p* = .58, with a small effect size (*Cohen’s d* = .04). Finally remission group also improves significantly, *t*(309) = 3.54, *p* = .00, with a small effect size (*Cohen’s d* = .22). These results are counterintuitive at first glance, one would expect a decrease in PMH for the incidence group, an increase in PMH for the remission group, and no change for the other two groups. However, all groups, except for the incidence group, improve significantly but the effect sizes are very small. At second glance, the Dresden sample is a non-clinical sample and only few expressed a wish for professional help [6]. Of the 1394 participants, 61 (4.4 %) women received psychotherapeutic treatment at baseline and 75 (5.4 %) at follow-up. On possible explanation is that the participants did not consider their overall well-being to be very strongly impaired. Therefore these findings are not so surprising after all.

The previous analyses illustrated that the PMH scale is sensitive to change at the aggregate level. This analysis does, however, ignore the effect of the unreliability of the test. If measurement error is present in the test score, then any observed change is an overestimate of the true change. In a clinical setting individual change is an important process parameter. In order to assess whether an individual’s change exceeds a change that can be expected due to the unreliability of the test, we use the Reliable Change Index (RCI). The reliable change index [25] can be considered a lower limit for clinical significant change. The RCI transforms the observed change into a standardized change score, taking measurement error into account. If this standardized change score exceeds 1.96 then there is a clinically significant change (p < .05). The RCI is computed from the test-retest reliability (*r*_*TR*_ = .81) and the standard deviation (*SD*_*t1*_ = 0.64) of the PMH score at the first administration. In total 211 (17.1 %) out of the 1231 psychosomatic patients showed a clinically significant change. This low number is not an indication that the PMH is not a valid instrument. To be specific, Jacobson et al. [26] warned against the misuse of the RCI to validate new measures “the method is not intended for validating the sensitivity of outcome measures.” In conclusion, 17.1 % merely indicates the percentage of patients that improved beyond the unreliability of the PMH scale. This is not a large percentage; however, psychosomatic disorders are among the most frustrating mental disorders for clinicians to manage and also result in high levels of patient dissatisfaction (e.g. [24]).

## Discussion

The PMH-scale was originally developed by Lutz et al. (1992a, unpublished manuscript) in order to provide a brief, unidimensional and person-centred instrument to assess positive mental health. In the present series of studies the PMH-scale was confirmed to be a unidimensional self-report instrument with high internal consistency, good retest-reliability, scalar invariance across samples and over time, good convergent and discriminant validity as well as sensitivity to therapeutic change in a series samples from very different backgrounds. The unidimensional structure of the PMH-scale suggests that it indeed measures a single concept. The equivalence tests indicate that the PMH-scale can be validly used to compare PMH-scale scores over groups and across time. The good test-retest reliability for the retest samples and the moderate correlation between the first and second measure for the psychosomatic patient group and Dresden sample suggests that the PMH-scale is both: stable over time and sensitive to change. With only nine items, the PMH-scale is brief and easy to interpret.

The present findings should be interpreted in light of the strengths and limitations of data collection. Particular strengths are that our study is based on several large, diverse samples from various areas of life, the prospective design in a community-based sample (Dresden sample) and the examination of validity of the PMH-scale with a broad range of questionnaires. Several limitations should also be noted. The fact that the participants for the first retest sample were recruited from the surroundings of the first author due to data availability, may have given rise to sample selection bias [20]. Interestingly, the results of the two other, very large representative samples used for assessing retest reliability were not much lower. Because the patient sample consisted of psychosomatic patients, the results on therapeutic change cannot be generalized to other mental health outpatients. In addition, for addressing the scale’s sensitivity to change (study 5) we do not have a comparison condition (such as a waitlist-control group, or a placebo control group). Furthermore, the data was collected twelve years ago. This means, that the diagnoses were based on the criteria of ICD-9, an older diagnostic tool for health assessment. Further studies should include more patient samples with a clear diagnosis based on more current diagnostic tools. The Dresden sample consisted of well-educated young women with a predominately medium to high socioeconomic status, which of course is not necessarily true for other populations. Nevertheless, the fact that the PMH-scale proved to be an important predictor for remission of phobias as assessed by state-of-the-art DSM-IV diagnoses is an argument for its potential usefulness in epidemiologic and clinical research. It may be beneficial for mental health care to focus on psychopathology and its treatment but also on promotion of positive mental health. Examples of mental health promotion in health care are well-being therapy [13] and Acceptance and Commitment Therapy [19], both psychotherapeutic approaches for increasing well-being. The PMH-scale can be used to examine the improvements in mental health. Patients may complete the PMH-scale at baseline and at regular intervals or the end of an outpatient or inpatient treatment.

## Conclusions

The PMH-scale is a good instrument for assessment of PMH in community and mental-health care. The PMH-scale provides a quick overall assessment of PMH. Because of its scalar invariance across time and its sensitivity to change, the PMH-scale may also be used for determining the effect of therapeutic or medical treatment on PMH.

## Abbreviations

BDI: Beck’s depression inventory
CES-D: Center for Epidemiological Studies Depression Scale
DASS stress: anxiety, depression, depressions anxiety stress scale
DPS: Dresden Predictor Study
EQ-5D: health status
F-DIPS: research diagnostic interview for psychological disorders
F-SOZU: social support scale
GKE: self-efficacy scale
LZH: life satisfaction scale
N-scale: neuroticism scale
PMH: positive mental health
PMH-scale: positive mental health scale
SHS: subjective happiness scale
SOC negative: sense of coherence negative items
SOC positive: sense of coherence positive items
SWLS: life satisfaction

## Footnotes

1 Mental health is a much contested area and there is no general agreement on terminology. For consistency, we will use the terms positive mental health (PMH) and mental disorder/mentally ill throughout this article.

2 More detailed information about reliability in the respective samples and the validity of the instruments can be obtained on request.
